# Intravaginal endoscopic vacuum therapy of a rectovaginal fistula: expanding boundaries

**DOI:** 10.1055/a-2505-9067

**Published:** 2025-01-16

**Authors:** Renato Medas, Guilherme Macedo, Eduardo Rodrigues-Pinto

**Affiliations:** 1 285211 Gastroenterology Department, Centro Hospitalar Universitário de São João, Porto, Portugal; 2 26706 Faculty of Medicine, University of Porto, Porto, Portugal

A 36-year-old woman underwent anterior resection of the rectum owing to endometriosis. The procedure was complicated by a rectovaginal fistula, which needed several surgical reinterventions. Despite diversion colostomy and reconstruction of the posterior vaginal wall with a fasciocutaneous flap, the rectovaginal fistula persisted, with a 50-mm abscess interposed between the coloanal anastomosis and the neovagina. The patient was referred for endoscopic evaluation.

Endoscopy confirmed a 4-mm fistulous tract at the coloanal anastomosis with communication between the abscess and vagina. Two sessions of endoscopic internal drainage were performed ( Fig. 1 **a** ); however, because of the lack of significant improvement in terms of the vaginal drainage and abscess dimensions, endoscopic intravaginal evaluation was performed, which revealed a 30-mm wide communication between the fornix and the abscess ( Fig. 1 **b** ).

**Fig. 1:**
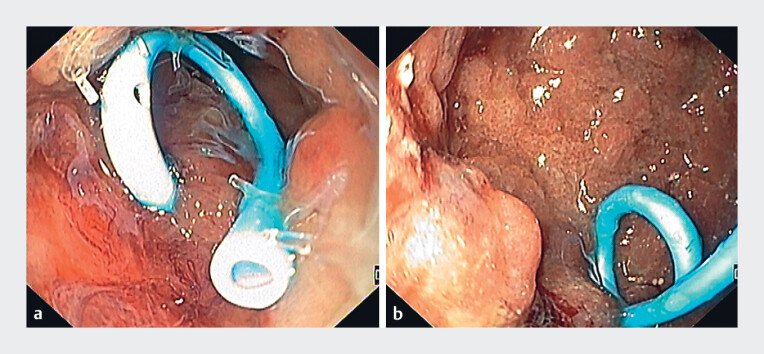
Endoscopic views showing: **a** endoscopic internal drainage of the abscess through the rectum; **b** the abscess cavity with a wide communication with the fornix on intravaginal view.

A total of seven sessions of intracavitary endoscopic vacuum therapy (EVT) were performed through the vagina ( Fig. 2 **a** ), each 3–4 days apart, with progressive reduction of the cavity size ( Video 1 ). Foreign bodies (surgical sutures/staples) were retrieved between the sessions to enhance tissue healing. At the end of the intravaginal EVT treatment, successful shrinkage of the cavity had been achieved, with a residual pseudodiverticulum ( Fig. 2 **b** ). Because of the persistence of contrast extravasation from the rectum to the pseudodiverticulum on rectal evaluation, a 12/6t over-the-scope (OTS) clip was placed on the rectal side ( Fig. 2 **c** ).

**Fig. 2:**
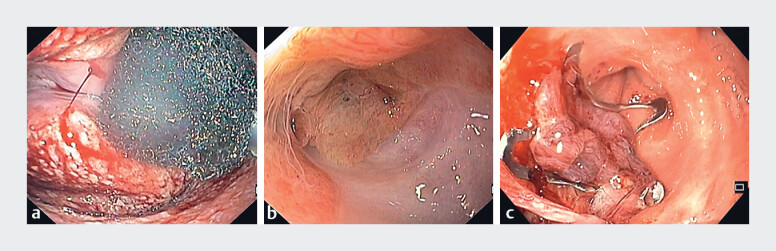
Endoscopic view showing: **a** the intravaginal endoscopic vacuum therapy; **b** the residual pseudodiverticulum after seven sessions of intravaginal endoscopic vacuum therapy had been completed; **c** an over-the-scope clip that was placed to close the fistulous tract between the rectum and the pseudodiverticulum.

Intravaginal endoscopic vacuum therapy is performed to close a rectovaginal fistula.Video 1

After 1 month, a double endoscopic evaluation (rectal and vaginal) was performed simultaneously, which showed OTS clip displacement, with no extravasation of contrast or methylene blue on either side. A computed tomography scan with rectal contrast and magnetic resonance imaging confirmed resolution of the fistula, with there being no recurrence during 12 months of follow-up.

Treatment of gastrointestinal fistulas frequently requires a multimodality approach, tailored to each phase of the healing process to enhance the possibility of clinical success 1 2 . To the best of our knowledge, this is the first report of intravaginal EVT, highlighting the expanding applications of EVT in the treatment of surgical complications.

Endoscopy_UCTN_Code_TTT_1AQ_2AG

## Notes

Endoscopy E-Videos https://eref.thieme.de/e-videos
*E-Videos* is an open access online section of the journal *Endoscopy* , reporting on interesting cases and new techniques in gastroenterological endoscopy. All papers include a high-quality video and are published with a Creative Commons CC-BY license. Endoscopy E-Videos qualify for HINARI discounts and waivers and eligibility is automatically checked during the submission process. We grant 100% waivers to articles whose corresponding authors are based in Group A countries and 50% waivers to those who are based in Group B countries as classified by Research4Life (see: https://www.research4life.org/access/eligibility/ ). This section has its own submission website at https://mc.manuscriptcentral.com/e-videos .
